# The regulation of pre-metastatic niche formation by neutrophils

**DOI:** 10.18632/oncotarget.22792

**Published:** 2017-11-30

**Authors:** Jadwiga Jablonska, Stephan Lang, Ronit Vogt Sionov, Zvi Granot

**Affiliations:** ^1^ Translational Oncology, Department of Otorhinolaryngology, University Hospital Essen, University Duisburg-Essen, Essen, Germany; ^2^ Department of Developmental Biology and Cancer Research, The Institute for Medical Research Israel-Canada, The Hebrew University-Hadassah Medical School, Jerusalem, Israel

**Keywords:** neutrophils, metastatic seeding, pre-metastatic niche, neutrophil polarization in tumor context

## Abstract

Metastasis is a multistep process requiring tumor cell detachment from the primary tumor and migration to target organs through the lymphatic or blood circulatory systems. Specific organs are predisposed to metastases in certain cancers and the formation of supportive metastatic microenvironment determines tumor cell homing. Such an environment is provided by a pre-metastatic niche that is formed through the recruitment of bone marrow-derived myeloid cells, however the mechanisms of its formation are not fully understood. Recent evidence suggests that the primary tumor itself modulates the environment of secondary organs prior to tumor cell dissemination. The contribution of neutrophils to the formation of the pre-metastatic niche is getting growing attention. Obviously, neutrophils can affect the development of metastasis in two contradicting ways, by either stimulation or inhibition of this process, depending on the activation status. Pro-tumor neutrophils actively support metastasis formation by different mechanisms, including the formation of pre-metastatic niche, tumor cell attraction, and the direct support of tumor cell proliferation. Moreover, suppressive neutrophils, which are the granulocytic arm of MDSC, promote tumor progression by dampening anti-tumor T cell immunity. On the other hand, anti-tumor neutrophils can inhibit metastasis formation by the cytotoxicity towards tumor cells in the circulation or at the pre-metastatic site, and even via stimulation of T cell proliferation. Apparently, the regulation of the pro- or anti-tumor neutrophil properties has significant implications on metastatic spread in the host. Here we provide an up to date overview of the different roles neutrophils play in regulating the metastatic processes.

## INTRODUCTION

Primary cancer can spread to distant organs in a process called metastasis. It is a multistep process that involves dissemination of tumor cells from a primary tumor, migration to a secondary site, invasion of the tissue and formation of metastatic nodules. Importantly, metastasis remains the primary cause of death in patients with solid cancers [1]. Growing primary tumors have been shown to influence the microenvironment of distant organs thus preparing them for the metastatic colonization. Many studies have demonstrated that specific organs are predisposed to metastases in certain cancers due to the formation of a supportive microenvironment in these organs [2]. Bone marrow derived myeloid cells, including neutrophils, have been shown to be the essential part of this process.

Neutrophils are the most abundant of all white blood cells and play a key role in host inflammatory responses. It is becoming increasingly clear that neutrophils possess various functions that dynamically regulate cancer progression and metastatic processes. These cells can exhibit either pro- or anti-tumor properties in a context dependent fashion. Pro-tumor neutrophils support angiogenesis via secretion or activation of pro-angiogenic factors. These cells mediate the proteolytic release of EGF, TGFβ and PDGF from the extracellular matrix (ECM). Neutrophils express also high levels of metalloproteinases, mainly MMP9, which can modify the ECM to allow tumor cell dissemination. Finally, certain neutrophils also possess immunosuppressive properties and are capable of limiting adaptive anti-tumor immune responses. This unique subpopulation of immunosuppressive neutrophils represents the granulocytic subset of myeloid derived suppressor cells (G-MDSC). Importantly, neutrophils have also been shown to possess anti-tumor properties, including the capacity to directly kill tumor cells through NETs formation or ROS release. Moreover, these cells are able to stimulate adaptive anti-tumor responses leading to the activation of T cells and tumor rejection.

The observation that neutrophils accumulate in large numbers in pre-metastatic organs [3, 4] has led to the hypothesis that neutrophils play a role pre-metastatic niche formation [5]. Similarly to the situation in primary tumors, these cells were shown to have either positive or negative effect on tumor cell seeding in the pre-metastatic site, depending on the cytokine landscape in the microenvironment [3, 6].

## NEUTROPHIL DEVELOPMENT AND REGULATION OF THEIR BONE MARROW RETENTION

Neutrophils are continuously produced in the bone marrow in a process termed granulopoiesis. During granulopoiesis, hematopoietic stem cells (HSCs) give rise to the multipotent common myeloid progenitors (CMPs) thereby committing to myeloid lineage differentiation. CMPs differentiate into bipotent granulocyte-macrophage progenitors (GMPs), which further differentiate into myeloblasts (MBs). Myeloblasts differentiate into promylocytes (PMs), myelocytes (MCs), metamyelocytes (MMs), and band cells (BCs). The final stage of neutrophil development is characterized by nuclear segmentation and the release of mature neutrophils into the circulation [7]. Mature neutrophils may remain within the bone marrow for 4–7 days [8, 9] and may constitute 90% of the mature neutrophil pool in the body, whereas only 1-5% of the neutrophil pool is found in the circulation [10, 11]. The large pool of mature neutrophils marginating in the bone marrow serves as a reservoir, which may be called when needed, and deployed immediately – a critical aspect of neutrophil function as first responders. To maintain this reservoir, the maintenance of neutrophils in the bone marrow needs to be tightly regulated. The retention of neutrophils in the bone marrow is mediated through interaction of CXCL12 (SDF-1; stromal-derived factor-1) with its receptor CXCR4 (CD184) [9]. CXCR4 is expressed during neutrophil differentiation and gradually disappears as the neutrophils mature [12]. This is mediated by G-CSF, which concomitantly drives neutrophil expansion and downregulates CXCR4 thereby promoting their release to the circulation [13]. The release of neutrophils from bone marrow is also supported by the up-regulation of CXCR2 on their surface [14]. The CXCR2/CXCR4 chemokine axis is also important for the trafficking of aged (senescent) neutrophils back to the bone marrow for clearance [8]. CXCR4 (CD184) expression is upregulated in aging neutrophils, whereas CXCR2 (CD182) is downregulated [15]. The upregulation of CXCR4 assists in sequestration of aged neutrophils back to the bone marrow, whereas downregulation of CXCR2 impairs neutrophil migration into inflammatory sites. In cancer, colony stimulating factors (G-CSF and GM-CSF) are often overexpressed leading to enhanced granulopoiesis and neutrophil release from the bone marrow [16, 17]. Of note, overexpression of colony stimulating factors it often associated with the release of immature neutrophils from the bone marrow, suggesting a skewing of neutrophil retention/release balance in the bone marrow.

### Molecular mechanisms of neutrophil activation and polarization during tumorigenesis

Neutrophil function in cancer has been a matter of debate as both tumor promoting and tumor limiting properties were documented. However, it seems that this debate was wrongly fueled by assumption that neutrophils are a homogeneous population of cells that may either promote or limit tumor growth and progression. We have come to realize that neutrophils come in various flavors - an understanding that is not too surprising when considering that these cells must be highly responsive to microenvironmental cues. As dictated by the cytokine milieu neutrophils may possess an anti-tumor N1 phenotype or a pro-tumor N2 phenotype. Further, neutrophils may be identified as have functions that on a wide spectrum that ranges between N1 and N2 [18–20]. N1 cells are cytotoxic, can eliminate tumors and are characterized with an immunostimulatory profile (i.e., TNFα^high^, CCL3^high^, ICAM-1^high^, Arginase^low^). Their cytotoxic activity is mediated by generation of high levels of superoxides and hydrogen peroxide, and they express higher levels of Fas, TNFα, CCL3, and ICAM-1 [21]. In contrast, N2 neutrophils are not cytotoxic, express a different repertoire of chemokines, and promote tumor growth as well as progression through the expression of high levels of immunomodulatory factors (i.e. Arginase) [22], angiogenic factors (i.e. VEGF and Bv8) [6, 23, 24], and matrix remodeling factors (i.e. MMP8 and MMP9) [25].

Several studies in recent years explored how neutrophil function is regulated in cancer and have shed light on the delicate balance that determines whether neutrophil act to promote or limit tumor growth and progression. Neutrophils are equipped with a wide range of receptors providing them with the ability to respond to a wide range of signals. For example, we have shown that various chemokines may stimulate a cytotoxic phenotype in resting neutrophils *in vitro* [3]. These chemokines were later shown to be secreted to various degrees in human cancers, leading to the generation of cytotoxic N1 neutrophils in patients. Another example is the role played by type I interferons (IFNs), which were initially characterized as potent anti- viral effectors. On top of the role IFNs play in immunity, they were found to possess potent anti-cancer activity. Moreover, IFNs were also found to promote anti-tumor immune responses via activation of the immune system. Initially, the effects of IFNs on anti-tumor immunity were limited to T-cells, NK cells and macrophages [26]. However, our studies in recent years have demonstrated that IFNs can also modify neutrophil function and promote neutrophil mediated anti-tumor immune responses (reviewed by Pylaeva et al. [27]). The consequences of IFN stimulation on neutrophil function were multifold: IFNs were found to suppress the pro-angiogenic activity of neutrophils [23], regulate neutrophil recruitment and life-span in the primary tumor [28, 29] and suppress the metastasis promoting activity of neutrophils in the pre-metastatic niche [6]. Similarly, TNFα was shown to limit tumor growth independently as well as though activation of anti-tumor immunity in neutrophils [30].

As mentioned above, numerous studies have shown that neutrophils in cancer also possess pro-tumor functions. Furthermore, neutrophil abundance in tumors is regarded as a poor prognostic factor. This suggests that although neutrophils stimulated by tumor-secreted factors have anti-tumor properties, the anti-tumor phenotype could be suppressed and an alternative, tumor-promoting phenotype is manifested. A groundbreaking study by Fridlender and colleagues demonstrated that TGFβ plays a critical role in modifying neutrophil function in the tumor microenvironment [31]. Specifically, blocking of TGFβ signaling induced massive accumulation of tumor-associated neutrophils (TAN). More importantly, unlike TAN in untreated tumors, which possess an N2 tumor-promoting phenotype, neutrophils recruited to the tumor in mice treated with a TGFβ signaling blocker possess an N1 anti-tumor phenotype. This suggests that TGFβ in the tumor microenvironment polarizes TAN towards an N2 pro-tumor phenotype. We and others published findings that strongly support these observations and show that TGFβ not only blocks neutrophil cytotoxicity [3] but also induces an immunosuppressive N2 neutrophil phenotype [19] and that TGFβ receptor deficient myeloid cells, including neutrophils, maintain an anti-tumor phenotype and limit tumor growth [32].

## THE PRE-METASTATIC NICHE HYPOTHESIS AND THE ROLE OF NEUTROPHILS IN THIS PROCESS

For successful metastatic colonization, tumor cells not only have to develop a specific genetic profile that enhances metastasis formation, but also need to modify the local microenvironment at the distant target organs [33]. Tumor-induced changes make tissues more receptive toward incoming disseminated tumor cells [2, 34]. Certain organs, such as the liver, lungs, brain, lymph nodes, and bone marrow are common sites of metastasis, while others, such as the kidney, pancreas and skin are rare [35].

Accordingly, before tumor cells arrive at the target organ, a so-called “pre-metastatic niche” formation is induced by factors derived from the primary tumor. The concept of pre-metastatic niche was proposed by Kaplan and colleagues in 2005 [5]. They could demonstrate that bone marrow-derived hematopoietic progenitor cells, which express vascular endothelial growth factor receptor 1 (VEGFR1, Flt1) accumulate early in the pre-metastatic lung and form cellular clusters before tumor cell arrival. Blocking of VEGFR1 function using antibodies or by the removal of VEGFR1^+^ cells from the bone marrow of wild-type mice abrogated the formation of these pre-metastatic clusters and prevented the formation of metastases [5].

Recent studies have revealed that neutrophils make up the main cell population involved in formation of pre-metastatic niche [4, 6, 36]. These cells are recruited by Bv8, MMP-9, S100A8, and S100A9 [4, 37] and this process seems to be strongly dependent on granulocyte colony-stimulating factor (G-CSF) [4]. G-CSF mobilizes neutrophils and facilitates their homing at distant organs even before the arrival of tumor cells. Moreover, such G-CSF-mobilized cells produce Bv8, a protein involved in activation of angiogenesis. Anti-G-CSF or anti-Bv8 treatment significantly reduced lung metastasis and number of circulating CD11b^+^Gr-1^+^ cells [4].

Hiratsuka and colleagues showed that primary tumor cells release VEGF-A, TGFβ and TNFα that in turn, induces the expression of the chemoattractants such as S100A8 and S100A9 by lung endothelium and myeloid cells [38]. Moreover Bv8 and metalloproteinase-9 (MMP9) are expressed in the pre-metastatic lung parenchyma and facilitate tumor cells homing [39]. Accordingly, Huh et al. demonstrated enhanced retention of human melanoma cells in lungs as early as 24 hours after co-injection with neutrophils into nude mice [40]. Formation of pre-metastatic niche by neutrophils seems to be enhanced by the absence of type I interferons. In such mice neutrophil trafficking into pre-metastatic lung is elevated due to the upregulated CXCR2 expression [6]. These neutrophils contribute to the elevated levels of pro-metastatic molecules like S100A8, S100A9, Bv8, and MMP9 in the lungs, supporting tumor cell homing to this organ [6]. The accumulation of neutrophils in pre-metastatic organs resembles the swarming activity of neutrophils, described in injury. Such swarming is stimulated by leukotriene B4 (LTB4) that is released by neutrophils. LTB4 is a lipid product of the arachidonate 5-lipoxygenase (ALOX5) enzyme [41] and its production by neutrophils in the pre-metastatic niche was shown to support LTB4 receptor (LTB4R) expressing “metastasis-initiating cells” in the MMTV-PyMT mouse model. Also, inhibition of ALOX5 reduced pulmonary metastasis without affecting primary tumor growth [42].

## RECRUITMENT OF NEUTROPHILS INTO PRE-METASTATIC SITES

Neutrophils make up significant proportion of the cells infiltrating tumors and the pre-metastatic niche in both mice and humans. The overall migration of neutrophils depends on chemotactic factors. There are several chemotactic factors involved in this process, but the most potent are members of the CXCL chemokine family. The most studied chemokine axis in the neutrophil context is the CXCR4/CXCL12 axis. It plays obviously role not only in BM retention of these cells, but also during migration of neutrophils into primary [23] and secondary tumor sites [43]. Notably, pre-metastatic sites such as lungs, bone, liver, brain and lymph nodes express high levels of CXCL12 [44]. *In vivo* inhibition of CXCR4/CXCL12 interactions significantly reduces metastasis of breast tumor cells to the lymph node and lungs [44].

Human CXCL8 (IL-8) is one of the best studied neutrophil chemoattractants with respect to human tumor biology. CXCL8 is overexpressed in different human carcinomas and tumor cell lines, such as breast, colon, cervical, lung, brain, prostate, ovarian and renal cell carcinomas, acute myelogenous and B-cell lymphocytic leukemia, melanoma and Hodgkin’s disease [45]. Of note, both tumor and stromal cells produce CXCL8. Another human chemokines, e.g. CCL3 (MIP-1α) and CXCL6 (huGCP-2) or murine chemokines CXCL1, CXCL2 and CXCL5, are potent chemoattractants and activators of neutrophils, and are released from many tumors [28, 46]. Recently TNFα-activated mesenchymal stromal cells were shown to promote breast cancer metastasis by recruiting neutrophils in CXCR2 dependent manner [47]. Another study on hepatocellular carcinoma indicated importance of CXCL16 and its receptor CXCR6 in neutrophil recruitment and tumor progression due to its ability to stimulate the release of CXCL8 by tumor cells [48]. Another recent study showed that human metastatic melanoma cells entrapped in the lungs secrete CXCL8 to attract neutrophils, which promotes tumor cell tethering to the vascular endothelium. Prolonged cell retention in the lungs facilitated trans-endothelial migration and metastasis development [40]. The inhibition of neutrophil migration by blocking of chemokine receptor CXCR2 or in CXCR2 deficient mice was shown to reduce tumor angiogenesis and growth in B16F10 melanoma [28] and Lewis lung carcinoma mouse models [49]. Inhibited myeloid cell infiltration due to the loss of CXCR2 was also shown to be responsible for suppressed chronic colonic inflammation, colitis-associated tumorigenesis [50] and reduction of metastasis in murine breast cancer tumor model [47]. CCL2 was also shown to play a role in migration of neutrophils during inflammatory processes [51, 52]. Tumors release high amounts of this chemokine [53], and the neutrophils from pre-metastatic lung up-regulate its ligand CCR2 [3]. However, the function of this cytokine in this context is not clear since it seems to rather stimulate neutrophil anti-metastatic capacity [3].

Additional mediators, besides chemokines, may serve as chemoattractants for neutrophils. Lipids, such as sphingosine-1-phosphate (S1P), were shown to promote neutrophil activation and chemotaxis [54]. Likewise, the hypoxia-inducible factor 1-α and its downstream products, such as CXCL12, VEGF or MMP9 are involved in the recruitment and retention of neutrophils in angiogenic environments [23, 55]. VEGF is also capable of inducing neutrophil adhesion to postcapillary venules resulting in efficient homing of these cells to tissues with high VEGF expression such as the primary tumor or the pre-metastatic niche [56]. The myeloid-related proteins (MRPs) are also involved in neutrophil migration. MRPs such as S100A8 or S100A9 are strongly expressed in the pre-metastatic niche and act as potent chemoattractants for neutrophils [6]. However, the exact mechanism of MRPs mediated neutrophil mobilization is not clear to date. Recently, the involvement of tumor exosomal RNAs in the formation of the lung pre-metastatic niche via TLR3 activation was demonstrated [57]. Authors could show that TLR3 signaling in lung epithelial cells induces chemokine secretion in the lung and boosts in turn the neutrophil recruitment. Neutrophils, which accumulate in the premetastatic niche, can alter their polarization state in the tumor-bearing host, switching from suppressing to promoting roles in tumor metastasis [57]. Tumors release exosomes into the surrounding microenvironment as well as into the bloodstream. The number of secreted exosomes correlates with the malignancy of the tumor [58] and has been suggested as potential mediator of pre-metastatic niche formation [59]. The mechanism of how exosomes should modify the micromilieu of pre-metastatic organs remains largely unknown; nonetheless, exosomes from melanoma cells have been described to reprogram bone marrow progenitor myeloid cells through the Met tyrosine kinase receptor, promoting a pro-angiogenic phenotype thus leading to the vascular leakiness at pre-metastatic sites [59].

## THE PRO-METASTATIC ROLE OF NEUTROPHILS

Neutrophils have been traditionally considered as the immune system first line of defense. However, in the context of cancer the function of these cells is frequently modified to act against the host and promote tumor growth as well as metastasis formation. Along these lines, several studies have shown that infiltration of tumors by neutrophils is associated with poor clinical outcome. Neutrophils have been shown to promote metastasis via a variety of mechanisms including induction of inflammation at secondary sites, promotion of tumor cell invasion and proliferation, extracellular matrix remodeling, angiogenesis and immune suppression.

### Inflammation as a stimulus for metastasis

Tumors were described as wounds that never heal [60]. Apparently, inflammation is strongly associated with primary tumor progression. However, the role of inflammation in metastasis formation is much less explored. It is well established that in the pro-metastatic stage neutrophils secrete soluble pro-inflammatory factors. Such factors, including proteases and cytokines that activate endothelium and parenchymal cells, lead to improved adhesion of circulating tumor cells in distant sites [61, 62] and to augmented metastasis formation. Neutrophils are capable of producing a number of cytokines including TNFα, IL-12 and CXCL8. Notably, CXCL8 is closely linked to NETosis and angiogenesis [63]. Recently, Rayes and colleagues demonstrated that inflammation in the lungs supports the recruitment of bone marrow-derived neutrophils which proteolytically destroy the anti-tumorigenic factor thrombospondin-1 (Tsp-1) [64], increasing metastatic progression. Accordingly, genetic ablation of these neutrophil proteases protected Tsp-1 from degradation and suppressed lung metastasis. In the recent work Wu et al. described that the lack of endogenous type I IFN signaling drives neutrophils to pro-metastatic phenotype. Such neutrophils infiltrate primary tumor and secondary organs, support the formation of the pre-metastatic niche, and show inhibited cytotoxicity against tumor cells [6].

### Direct neutrophil-tumor cell contact

Contact-dependent mechanisms where neutrophils tether circulating tumor cells (CTCs) to target-organ endothelium have been described [65]. Such interaction is mediated by the β2 integrins on neutrophils that bind to ICAM-1 on tumor cells and was described for liver and lung metastasis model [40, 66]. Spicer et al. showed that neutrophils promote cancer cell adhesion within liver sinusoids and their depletion before cancer cell inoculation resulted in decreased number of metastases in an intra-splenic model of liver metastasis [66]. Another study revealed that neutrophils interact with circulating tumor cells and anchor them to the endothelium, thus promoting lung metastasis [40].

### Interaction of neutrophils with activated endothelial cells supports metastasis

Vascular endothelial cells (ECs) compose tumor microenvironment and play important role not only in blood vessel formation, but also in production of pro-tumor molecules, and in the recruitment of immune cells such as neutrophils. Therefore, activation of ECs would be important in the regulation of metastasis formation. Wieland et al. have shown recently that activated Notch1 signaling in ECs could participate in metastasis formation via up-regulation of VCAM-1 expression on these cells and the expression of neutrophil attracting chemokines. This in turn results in infiltration of neutrophils [67]. Blockade of Notch1 or VCAM-1 reduces infiltration of neutrophils and resulting lung metastasis formation in mice [67]. Activation of ICAM-1 expression on endothelial surfaces in tumor was also shown to play a role in metastasis formation [68]. Elevated ICAM-1 expression promotes release of neutrophil specific chemoattractants. Adherence between neutrophil cell surface ligands and tumor-expressed ICAM-1 stimulates neutrophil degranulation releasing elastases, which break down endovascular and endolymphatic barriers permitting transendothelial tumor cell migration [68].

### Neutrophil-derived leukotrienes promote metastatic niche formation

Metastatic seeding requires not only a competent seed but also a fertile soil [69]. In 2005, Kaplan and colleagues showed that VEGFR1^+^ bone marrow derived cells accumulate in the pre-metastatic organ and prime a pre-metastatic niche that is more receptive for disseminated tumor cells [5]. While neutrophils are in fact VEGFR1^+^, they are not the only bone marrow derived cells that accumulate in the pre-metastatic niche, and other VEGFR1^+^ cells, such as monocytes, may be responsible for priming of the pre-metastatic niche [48]. A more precise evidence for the role neutrophils play in promoting metastatic niche formation is associated with the activity of neutrophil derived leukotrienes. Specifically, neutrophil secreted leukotriene was found to induce both tumor cell proliferation and metastatic efficiency [42].

### The role of NETs in metastatic processes

Production of extracellular traps (NETs) is an important feature in neutrophil biology. Such NETs are composed of chromatin fibers decorated with histones and other proteins, and are considered as an additional tool in neutrophils’ anti-microbial arsenal, but also as possible mechanism that contributes to cancer progression and metastasis. NETs are capable of trapping circulating tumor cells and depending on neutrophil activation such sequestered tumor cells can be destroyed by ROS, resulting in the inhibition of metastasis formation [6], or be kept in place thus supporting early adhesion of tumor cells to distant organ sites [70]. There are many factors that can induce NETosis. CXCL8 has been shown to promote the formation of NETs by activated neutrophils in preeclampsia patients [71]. NETs can also be induced within the vasculature in experimentally induced surgical stress thus supporting the metastatic seeding of cancer cells in the liver [72]. Severe post-surgical infection is also known to be associated with enhanced NET formation and support of metastasis. Cools-Lartigue et al. could show that septic mice that had undergone caecal ligation and puncture develop microvascular NETs that efficiently trap circulating lung carcinoma cells. This ultimately leads to the higher metastatic burden in lung. This effect could be abrogated by degradation of NETs by DNase I [70]. Recently, circulating breast cancer cells were shown to induce neutrophils to release NETs [73]. Such NETs contributed to the enhanced cancer cell migration and invasion of target organs. Of note, treatment with NET-digesting DNase I-coated nanoparticles was shown to inhibit metastasis in this experimental setting [73].

### Neutrophils suppress adaptive immune responses to facilitate metastasis

Luckily, metastatic progression is a highly inefficient process where only a minute fraction of disseminated tumor cells will eventually form distant tumors. While this is often a result of tumor cell autonomous properties (invasiveness, motility, etc.), metastatic progression is prevented by anti-tumor immune responses directed against disseminated tumor cells. That said, tumors induce the propagation of immunosuppressive myeloid cell populations which have the capacity to limit anti-tumor adaptive immunity to promote metastatic spread. For lack of a better terminology, these immunosuppressive myeloid cell populations were collectively termed myeloid derived suppressor cells (MDSC). MDSC can roughly be divided into mononuclear (M-MDSC) and granulocytic (G-MDSC).G-MDSC are characterized as a CD11b^+^Ly6G^+^Ly6C^lo^ cells in mice [74, 75]. Importantly, mature mouse neutrophils are also CD11b^+^Ly6G^+^Ly6C^lo^ making it impossible to distinguish between neutrophils and G-MDSC. The definition of G-MDSC in humans is even more complex and several phenotypes were suggested including: CD15^+^CD66^+^CD33^dim^HLA-DR^-^ [76], CD33^+^CD11b^+^HLA-DR^low/-^CD15^+^ [77] and CD11b^+^CD14^-^HLA-DR^low/-^CD33^+^CD15^+^ [78, 79]. As in the mouse, these human G-MDSC markers do not provide a clear distinction between G-MDSC and neutrophils. Furthermore, the nature of these cells is not completely defined as they consist of low-density neutrophils, immature neutrophils or mature neutrophils, with one common denominator - they all display immunosuppressive functions [80]. Such immunosuppressive or regulatory neutrophils contain large amounts of arginase I which suppresses T cell proliferation through deprivation of L-arginine [81, 82]. Another mode that immunosuppressive neutrophils utilize to block T-cell proliferation is the generation of high levels of hydrogen peroxide [74, 83]. The plethora of studies investigating the role of immunosuppressive neutrophils (G-MDSC) have demonstrated their contribution in multiple aspects of tumorigenesis. These neutrophils were shown to contribute to the formation of an immunosuppressive microenvironment at the primary tumor, leading to T-cell exhaustion and enhanced tumor growth [19, 84]. Moreover, unlike cytotoxic neutrophils which actively act to limit metastatic seeding, non-cytotoxic immunosuppressive neutrophils limit anti-tumor immune responses and enhance metastatic seeding in distant sites [85].

### THE ANTI-METASTATIC ROLE OF NEUTROPHILS

Anti-tumor neutrophils (N1) act to limit not only tumor growth but also metastatic progression. This is accomplished via diverse mechanisms including inflammation, direct and antibody dependent cytotoxicity as well as through the activation of other cell types including T-cells or dendritic cells.

### Inflammation

Type I interferons have been shown to be potent suppressors of tumor growth and metastasis. The role of interferons in polarization of tumor associated neutrophils has been recently reviewed in length by Pylaeva et al. [27]. Interferons suppress the formation of pre-metastatic niche via down-regulation of neutrophil migration into the lungs and their survival there [6]. Moreover interferon down-regulates the expression of VEGF and MMP9 on the gene and protein level [23, 86] and suppresses S100A8 and S100A9 [6]. Interferons increase the neutrophil cytotoxicity against tumor cells via enhancement of ROS production and boosted NETs release [21]. Tumor cells trapped in NETs can be efficiently killed via ROS or other reactive species secreted from the granules of activated neutrophils. Interferons also are known to increase adaptive immune responses via up-regulation of antigen presentation and efficient T cell stimulation. It should be noted that a link exists between generation of ROS and neutrophil ER stress and increased neutrophil ER stress was shown to exacerbate tissue damage in acute lung injury [87]. While ROS production is linked to ER stress and could potentially kill tumor cells, induction of ER stress in neutrophils can also induce an immunosuppressive phenotype and promote immune evasion [88].

### Neutrophil cytotoxicity

Direct cytotoxicity of neutrophils towards tumor cells was first observed in the early 1970’s [89]. It is mainly mediated by a variety of anti-microbial molecules generated by neutrophils. Most of these molecules are directed against bacterial pathogens thus not harmful for the eukaryotic cells [7]. Nevertheless, neutrophils can also produce reactive factors *i.e.* superoxides, H_2_O_2_ and HOCl that can destroy tumor cells [90]. It was shown that physical contact is required for neutrophil cytotoxicity. For such direct cytotoxicity a direct cell-cell contact is needed that can be provided e.g. by the NET trapping of the tumor cell. However, stimulating cultured neutrophils with a potent agonist, such as PMA, leads to the generation and secretion of very high levels of H_2_O_2_ alleviating the need for physical contact [3]. Another form of cytotoxicity exerted by the neutrophils is an antibody-dependent cell-mediated cytotoxicity (ADCC). Neutrophils express several FcRs that can mediate ADCC, including FcγRI (CD64), FcγRIIa (CD32), FcγRIIIa (CD16a), and FcγRIIIb (CD16b) [91, 92]. Indeed, neutrophils were shown to take part in ADCC in several types of cancer including glioma, squamous cell and ovarian carcinoma, breast cancer [93], Non-Hodgkin’s Lymphoma [94] and in B-cell lymphoma [95].

### Stimulation of adaptive immune system

The immune regulatory functions of neutrophils are recently getting attention. Apparently, the interactions between neutrophils and other immune cells regulate many inflammatory processes, including tumorigenesis. There is evidence that activated neutrophils can interact with T cells in manifold ways. They are required for proper anti-tumor CD4^+^ and CD8^+^ T-cell responses [31, 96–98] as they were shown to present antigens and provide accessory signals for T cell activation [99, 100]. Of note, until recently antigen presentation was thought to be exclusively mediated by dendritic cells (DC). Nevertheless, in 2007 Beauvillain and colleagues could demonstrate that neutrophils efficiently process and present antigens to T-cells [101]. While this does not directly link neutrophil presentation of antigens to anti-tumor cytotoxicity, Fridlender et al. showed in 2009 that N1 TAN require T-cells for their anti-tumor activity in the primary tumor [31]. This phenomenon could be explained by neutrophils capacity to stimulate T-cells via tumor antigens presentation. Additionally, neutrophils are able to recruit and activate T-cells via secretion of cytokines, including TNFα, Cathepsin G and neutrophil elastase, which have a direct effect on T-cells and promote their proliferation and cytokine production [84]. This leads to overall stimulation of the adaptive immune anti-tumor responses.

### Neutrophil extracellular traps formation

Neutrophil extracellular traps (NETs) can support metastasis processes, but can be also involved in anti-metastatic responses of neutrophils as they can be exploited to prime T-cells. NETs released by neutrophils can directly prime T cells by reducing their activation threshold. NETs-mediated priming increases T cell responses to specific antigens and even to suboptimal stimuli, which would not induce a response in resting T cells [102]. Furthermore, NETs can modulate immune responses by activating plasmacytoid dendritic cells (pDCs) via TLR9 [103]. Evidently, NETs influence an immunoediting of cancer and therefore support anti-tumor immune responses [104]. Depending on their activation status, neutrophils can kill disseminated cancer cells [3]. Trapping into NETs provides closer contact with cancer cell thus supporting direct killing by activated neutrophil. This scenario could be an explanation for the phenomenon observed in 4T1 tumor bearing mice, where enhanced NETs release together with high ROS expression by neutrophils resulted in reduced number of metastasis in the lung [6]. In contrast, in mice deficient for type I IFNs the release of NETs and ROS is significantly impaired, leading to enhanced metastasis in the lung of such mice [6].

### CONCLUDING REMARKS

**Figure 1:**
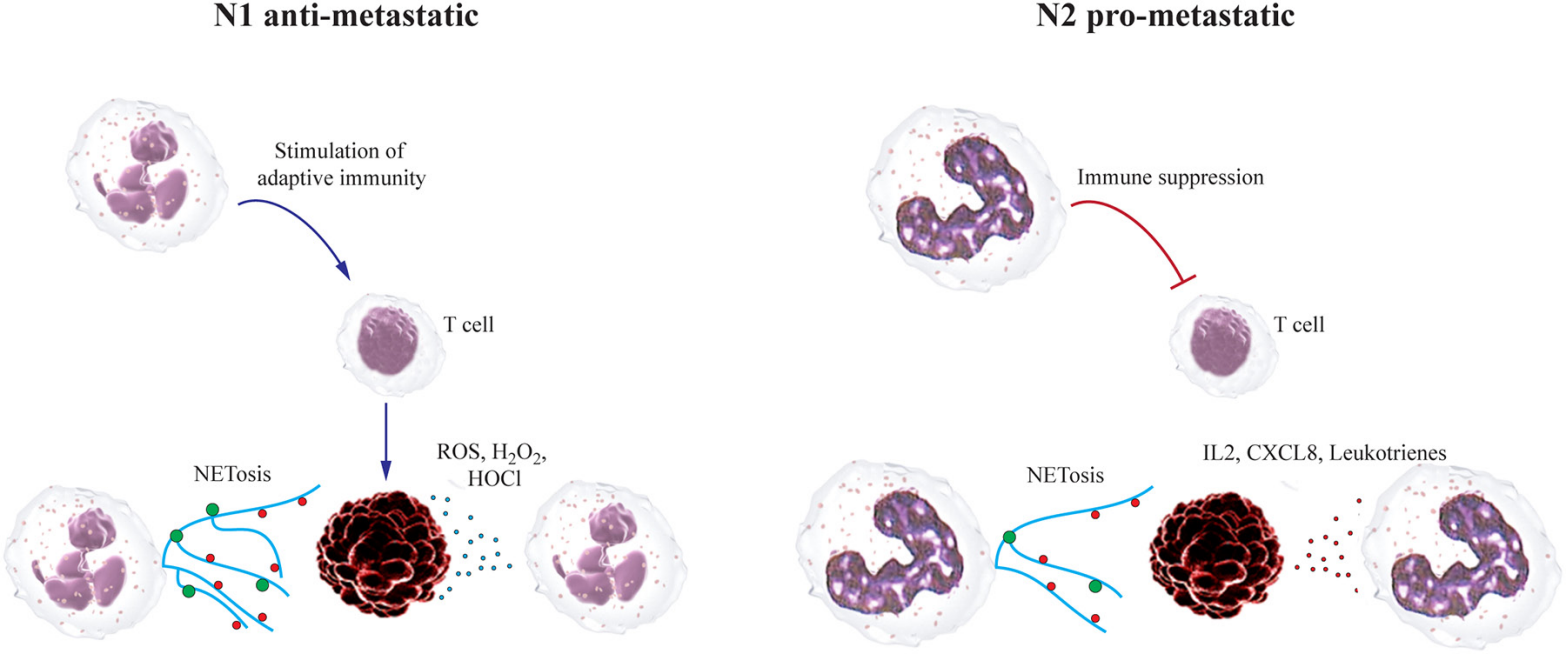
Neutrophils can play dual roles during the establishment of metastatic niche Anti-metastatic neutrophils release NETs, ROS and efficiently kill tumor cells already in the circulation. They do not form efficient pre-metastatic niche. As a result metastatic load in the lungs are diminished. Pro-metastatic neutrophils have suppressed cytotoxicity against tumor cells but high migratory and pro-angiogenic capacity. They migrate into lungs and form pre-metastatic niche that attracts circulating tumor cells. As a result metastases are formed.

**Figure 2:**
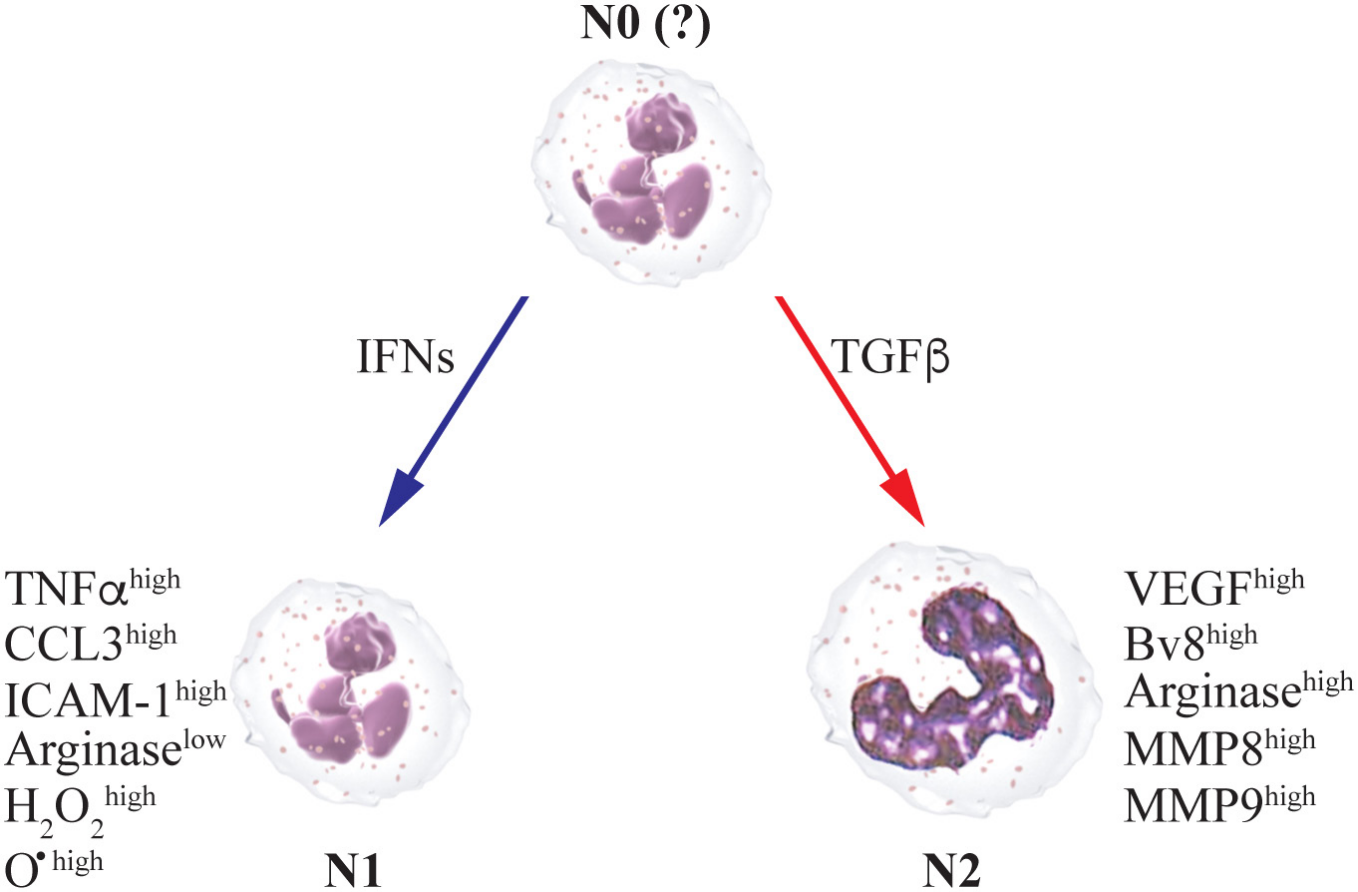
The anti- or pro-metastatic functions of neutrophils depend on the cytokine milieu in the microenvironment N1 anti-metastatic state is supported by type I IFNs, while TGFβ primes N2 pro-metastatic polarization

In the process of oncogenesis neutrophils exhibit a range of tumor promoting as well as tumor limiting properties. Importantly, these cells regulate not only primary tumor growth, but can also mediate the formation of the so called “pre-metastatic niche” (Figure 1). Together, these observations once again support the concept that neutrophil function in cancer situation depends on the activation status of these cells, which is regulated by manifold of tumor-released factors such as IFNs or TGFb (Figure 2). This review identifies potential elements that could be therapeutically targeted to enhance anti-tumor activity of neutrophils while restricting their pro-tumor characteristics.
